# Parliament and the Profession

**Published:** 1918-05-25

**Authors:** 


### PARLIAMENT AND THE PROFESSION.

### Gratuities to Territorial Force Medical Officers.

IN reply to a question by Sir Watson Cheyne, Mr. Forster said that the gratuity provided in the pay warrant for Territorial Force officers of all kinds is not payable until completion of service. In the case of the temporary medical officer the terms of the contracts provide for the payment of an annual gratuity.

### Hospital Scrubbers and the War Council.

MR. JOWETT called the attention of the Financial Secretary to the War Office to the position of the scrubbers in St. Luke's War Hospital, Bradford, where work was begun at 7 A.M. and continued until 5 P.M. with one and a-half hour interval for meals. Sunday work is not less than two hours, but five hours' holiday are given each week, the weekly wage being 18s., with no meals provided. Mr. Forster replied that authority has already been given for the rate of pay to be temporarily increased to 22s. per week.

### Masseuses in Military Hospitals.

MR. R. GWYNNE asked the Financial Secretary to the War Office whether there is any reason why masseuses who work in military hospitals are paid £2 10s. a week while those in naval hospitals receive £3 a week. Mr. Forster replied that representations have been received, and are now under consideration.

### Inoculation.

MR. MACPHERSON informed Sir A. Black that soldiers are now inoculated with a vaccine which has for its object protection against typhoid fever and paratyphoid A and paratyphoid B. Two doses of the vaccine are administered, the second being given after an interval of ten days. Soldiers are re-inoculated yearly.

